# Glucagon-like peptide-1 receptor agonists and the risk of Parkinson’s disease in patients with type 2 diabetes: a systematic review and meta-analysis of large-scale real-world data

**DOI:** 10.3389/fendo.2026.1849318

**Published:** 2026-08-13

**Authors:** Ying Chen, Xueping Wang, Jingjuan Wang, Gaiping Ma

**Affiliations:** 1 Department of Neurosurgery, Daping Hospital, Army Medical University, Chongqing, China; 2 Department of Neurology, Daping Hospital, Army Medical University, Chongqing, China

**Keywords:** glucagon-like peptide-1 receptor agonists, meta-analysis, Parkinson’s disease, real world study, systematic review

## Abstract

**Background:**

Parkinson’s disease (PD) is the second most common neurodegenerative disorder, yet effective disease-modifying therapies remain elusive. Glucagon-like peptide-1 receptor agonists (GLP-1RAs), widely used in the management of type 2 diabetes mellitus (T2DM), have shown neuroprotective potential in preclinical studies. However, real-world evidence on the association between GLP-1RAs use and PD risk remains inconsistent and has not been systematically synthesized. This systematic review and meta-analysis aimed to evaluate this association using large-scale real-world data.

**Methods:**

We systematically searched PubMed, Embase, the Cochrane Library, and Web of Science from database inception to March 18, 2026, for cohort and case–control studies exploring the association between GLP-1RA exposure and PD risk among patients with T2DM. Study selection, data extraction, and methodological quality assessment were independently conducted by two reviewers. All meta-analyses were performed using Stata 15.0 software.

**Results:**

A total of six studies were included, involving 452,763 participants. The meta-analysis revealed that GLP-1RA exposure was significantly associated with a reduced risk of PD in patients with T2DM (HR = 0.68, 95%CI (0.54,0.85), P = 0.001). Subgroup analyses demonstrated that compared with dipeptidyl peptidase-4 (DPP-4) inhibitors, metformin, and other hypoglycemic drugs, GLP-1RAs were linked to a lower risk of incident PD (DPP-4 inhibitors: HR = 0.60, 95%CI (0.43,0.83), P = 0.001; Metformin: HR = 0.83, 95%CI (0.73,0.95), P = 0.006; Other: HR = 0.73, 95%CI (0.60,0.87), P = 0.002).

**Conclusions:**

This meta-analysis based on large-scale real-world data indicates that GLP-1RA exposure is associated with a lower risk of PD in patients with T2DM, with robust and consistent results across diverse comparator groups. These findings support the hypothesis that GLP-1RAs may confer neuroprotective properties, a finding that merits further rigorous investigation. Nevertheless, given the inherent limitations inherent to observational study designs and the possibility of residual confounding in the included studies, the present results should be regarded as preliminary and hypothesis-generating rather than conclusive. Future large-scale prospective cohort studies with standardized methodologies, as well as well-designed randomized controlled trials, are warranted to validate these findings and clarify whether the observed inverse association reflects a causal neuroprotective effect of GLP-1RAs.

## Introduction

Parkinson’s disease (PD) is the second most prevalent neurodegenerative disorder, secondary only to Alzheimer’s disease (1). Driven by global population aging, the prevalence of PD continues to rise steadily. By 2030, the total number of PD patients worldwide is projected to exceed 9 million (2). Pathologically, PD is characterized by progressive degeneration of dopaminergic neurons in the substantia nigra and abnormal aggregation of α-synuclein (3, 4). Its cardinal motor manifestations include resting tremor, rigidity, bradykinesia, and postural and gait disturbances (5). A spectrum of non-motor symptoms, including cognitive impairment, depression, and autonomic dysfunction, frequently coexist with motor deficits, severely compromising patients’ quality of life and imposing a substantial socioeconomic burden on families and healthcare systems globally (6–9).

Recent evidence indicates a plausible pharmacological association between neurodegenerative diseases and type 2 diabetes mellitus (T2DM) (10). Patients with T2DM exhibit a 38% higher risk of developing PD relative to nondiabetic individuals, and accumulating data support a link between α-synuclein, pathological features and insulin resistance (10, 11). Glucagon-like peptide-1 receptor agonists (GLP-1RAs) represent a novel class of glucose-lowering medications for the clinical management of T2DM that have entered clinical practice in recent decades (12). Their hypoglycemic mechanisms include promoting glucose-dependent insulin secretion, inhibiting glucagon release, delaying gastric emptying and reducing body weight, among other actions (13, 14). Notably, preclinical findings have increasingly uncovered the promising neuroprotective potential of GLP-1RAs in the central nervous system. GLP-1 receptors are widely distributed in multiple brain regions, including the substantia nigra, striatum, and hippocampus. Activation of the GLP-1R signaling cascade confers multifaceted neuroprotective benefits, including ameliorating mitochondrial dysfunction, mitigating neuroinflammation, attenuating α-synuclein aggregation, enhancing autophagic activity, and facilitating neurogenesis (15–17). Collectively, these preclinical mechanisms suggest that GLP-1RAs may hold potential to slow or halt the pathological progression of PD, rendering them promising candidate disease-modifying therapies for PD.

In recent years, numerous studies (18–23) have explored the association between GLP-1RA exposure and the risk of incident PD in patients with T2DM. Some studies (18, 20, 23) have shown that patients with T2DM treated with GLP-1RAs exhibit a significantly reduced risk of PD, while other studies (21, 22) failed to identify a statistically significant association. To date, no systematic collation and quantitative meta-synthesis of large-scale real-world datasets have been conducted. Accordingly, the overall direction and magnitude of the protective effect, along with the relative neuroprotective benefits of GLP-1RAs versus alternative glucose-lowering agents, remain inconclusive. Given this research gap, this systematic review and meta-analysis was designed to assess the inverse association between GLP-1RA exposure and PD risk in patients with T2DM by synthesizing eligible cohort and case–control studies, and further compare the protective effects of GLP-1RAs with dipeptidyl peptidase-4 (DPP-4) inhibitors, metformin and other glucose-lowering agents, aiming to generate credible evidence to guide clinical practice, facilitate drug repurposing, and inform the development of novel neuroprotective therapeutic strategies.

## Methods

This study was conducted in accordance with the updated Preferred Reporting Items for Systematic Reviews and Meta-Analyses (PRISMA) guidelines (24–26).

### Search strategy

We systematically searched four electronic databases, including PubMed, Embase, the Cochrane Library, and Web of Science, from database inception to March 18, 2026. Eligible cohort and case–control observational studies focusing on the association between GLP-1RA exposure and the risk of incident PD in patients with T2DM were retrieved. No language restrictions were imposed. A comprehensive literature search was performed using a combination of Medical Subject Headings (MeSH) terms and free-text terms. Additionally, the reference lists of all included articles were manually screened to identify potentially eligible unpublished or overlooked publications. The search terms included but were not limited to: “Glucagon-Like Peptide-1 Receptor Agonists”,”GLP-1 Receptor Agonists”,”GLP-1RAs”,”GLP-1 Analogs”, Parkinson Disease”, “Parkinson’s Disease”, “Diabetes Mellitus, Type 2”,”Type 2 Diabetes Mellitus”, “Type 2 Diabetes”, “Real-World Data”, “Real-World Evidence”, “Cohort Studies”, “Case-Control Studies”, “Observational Study”, among other search terms.

### Inclusion and exclusion criteria

Studies that fulfilled all of the following criteria were eligible for inclusion:

1. Study design: Cohort study or case-control study;
2. Participants: Patients with type 2 diabetes, with no restrictions on age, gender, or race or ethnicity;
3. Exposure factors: GLP-1RA exposure, including liraglutide, semaglutide, exenatide, dulaglutide, beinaglutide, and others;
4. Outcome: the incident of PD was defined as a newly diagnosed PD case during the follow-up period, confirmed by International Classification of Diseases (ICD) coding or standardized clinical diagnostic criteria;
5. Effect estimate: studies providing extractable hazard ratio (HR) with corresponding 95% confidence interval (CI).

Studies were excluded if they met any of the following criteria:

1. Duplicate publications;
2. Studies with insufficient raw data for quantitative effect estimation;
3. Non-original studies, including systematic reviews, narrative reviews, conference abstracts, case reports, case series, commentaries, letters, and preclinical animal studies.

### Data extraction

Study selection and data extraction were independently conducted and cross-checked by two researchers. Firstly, they excluded clearly irrelevant citations by reading the titles and abstracts. Then, full-text articles of the remaining eligible records were subsequently assessed for final inclusion. Any discrepancies between the two reviewers were resolved via consensus discussion, with arbitration by a third independent reviewer when necessary.

All relevant data were extracted using a standardized pre-designed data extraction form, covering the following key information:

1. Basic study characteristics: First author, publication year, country, study type;
2. Baseline participant characteristics: Sample size, age, gender composition, follow-up time;
3. Exposure and comparator information: Type of GLP-1RAs, comparator drug class;
4. Primary outcome data: Number of PD cases, effect value and its 95% CI;
5. Confounding covariates adjusted for in the primary statistical analysis;
6. Detailed risk-of-bias assessment results.

### Risk of bias

Two researchers independently evaluated the methodological quality of the included studies, and reached a consensus after cross-checking. The Newcastle-Ottawa Scale (NOS) was used to assess the quality of the included cohort studies and case-control studies. The NOS scale consists of three dimensions: Selection (4 points), Comparability (2 points), and Exposure/Outcome (3 points), with a total score of 9 points. According to the aggregate NOS score, included studies were categorized as high quality (7–9 points), moderate quality (4–6 points), or low quality (0–3 points).

### Statistical analysis

Meta-analyses were conducted using Stata 15.0 software. Pooled HR together with their corresponding CI were adopted as the summary effect size. P value < 0.05 was defined as statistically significant. The heterogeneity among the included studies was evaluated using the Cochran Q test and the I² statistic. When I² < 50% and P > 0.1, this threshold indicated a small degree of heterogeneity, and the fixed-effect model was used; when I² ≥ 50% or P ≤ 0.1, it indicated significant heterogeneity, and the random-effect model was used, followed by subgroup and sensitivity analyses to explore potential sources of heterogeneity. Subgroup analysis was performed based on the type of comparator drug class, and the effects of GLP-1RAs compared to DPP-4 inhibitors, metformin, and other hypoglycemic drugs (primarily sulfonylureas, insulin, thiazolidinediones, and meglitinides) on the risk of PD were evaluated. Sensitivity analyses were performed by sequentially excluding each individual study and rerunning the pooled meta-analysis to verify the robustness of the overall effect estimate and identify whether any single study exerted an undue influence on the pooled results. Publication bias was evaluated both visually through funnel plot asymmetry inspection and quantitatively using Egger’s linear regression test.

## Results

### Searching results

**Figure 1:**
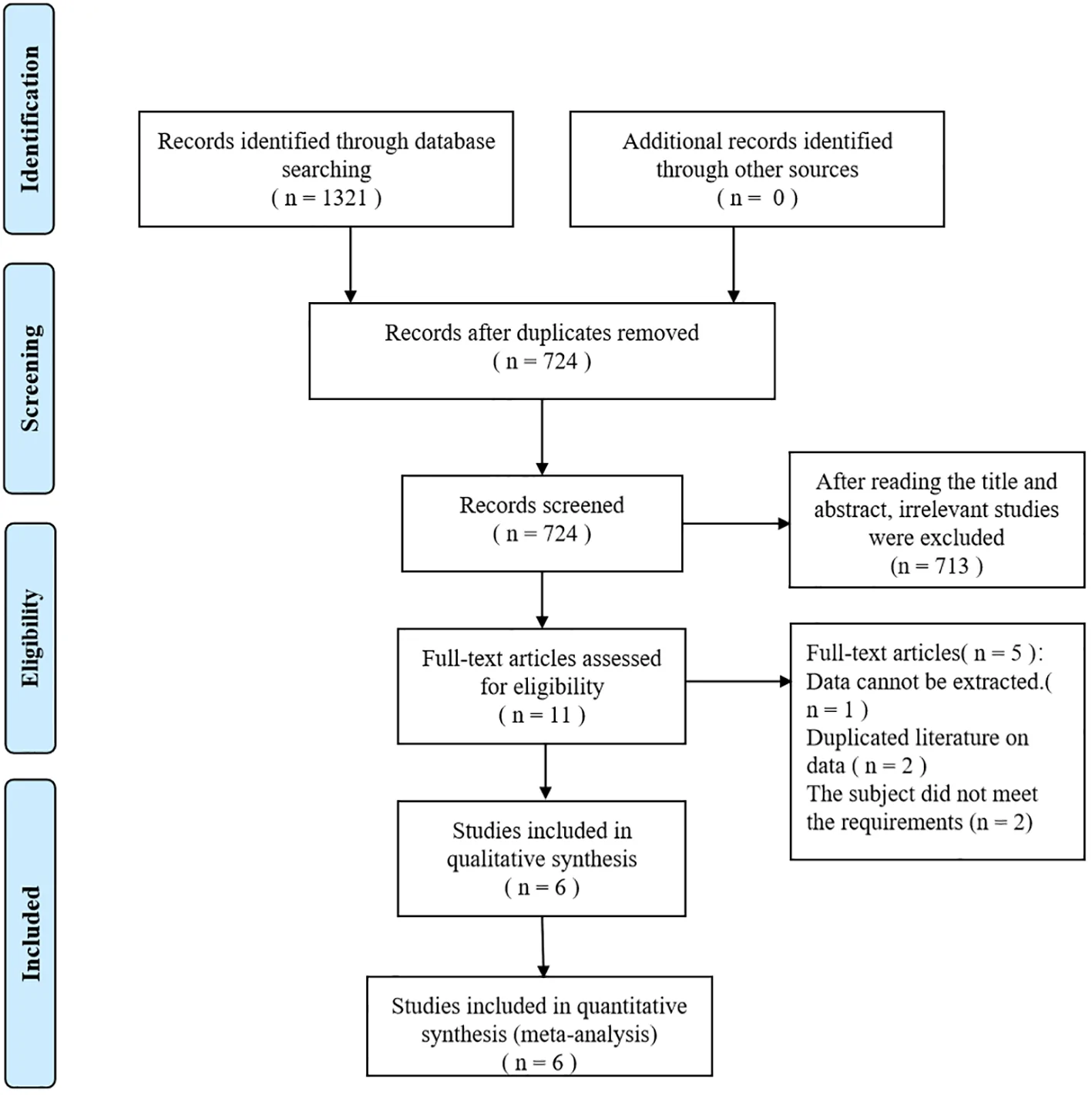
Flow chart of the study selection procedure. This diagram illustrates the systematic literature screening process following PRISMA 2020 guidelines. A total of 1,321 records were identified from four databases. After removing duplicates (n = 312), 1,009 records were screened by title and abstract, of which 968 were excluded. The remaining 41 full-text articles were assessed for eligibility, and 35 were excluded. Ultimately, six studies met the inclusion criteria and were included in the meta-analysis.

The initial systematic search across PubMed, Embase, the Cochrane Library, and Web of Science retrieved a total of 1,321 records. After duplicate removal, title and abstract screening, and full-text eligibility assessment, six qualified observational studies (18–23) were ultimately included in the meta-analysis. The final dataset comprised five retrospective cohort studies and one case–control study, involving a total of 452,763 participants (**Figure 1**).

### Characteristics of eligible studies

The detailed baseline characteristics of all included studies are summarized in **Table 1**. The six eligible studies were conducted across the United Kingdom, Denmark, the United States, Finland, and Israel, with several investigations based on multinational research networks. All datasets were extracted from large-scale real-world healthcare databases, including the UK Health Improvement Network, Danish National Health Registries, US Medicare administrative database, Finnish national registries, Israel’s Maccabi Healthcare Services database, and the TriNetX global research network. The study follow-up periods ranged from 1999 to 2025. All enrolled participants were patients with T2DM, with sample sizes varying from 9,951 to 184,970 across individual studies. The mean or median participant age ranged from 58.7 to 75.1 years, and the overall study populations exhibited a slight male predominance. Diagnoses of PD were ascertained based on clinical medical records, ICD coding, neurologist confirmations, and/or prescriptions for anti-Parkinson medications. The follow-up duration of each study varied widely, ranging from 1.54 to 20 years. All primary analyses were adequately adjusted for core confounding factors, including age, sex, baseline comorbidities, and concomitant medications. Methodological quality assessment revealed that the NOS scores of included studies ranged from 7 to 8 points, indicating high overall study quality (**Table 2**).

**Table 1:** Characteristics of included studies.

Study	Country	Database source	The period of the study	Study design	Patients(n)	Sex (Male/Female)	Age (Median/Mean), years	Parkinson’s disease diagnostic criteria	Follow-up	Adjustment factors
Brauer et al., 2020 (18)	UK	The Health Improvement Network	2006–2019	Retrospective cohort study	49077	28687/20390	60.6	The first recorded diagnosis of Parkinson’s disease in the clinical record	GLP-1RAs:3.6 years/comparison group:2.81 years	Age, gender, smoking status, alcohol consumption, BMI, duration of diabetes, HbA1c, comorbidities, use of calcium channel blockers
Gamborg et al., 2025 (19)	Denmark	Danish nationwide health registries	2007–2018	Retrospective cohort study	33462	19385/14077	62.5/62.4	Diagnosis of Parkinson’s disease based on registration	Patients enrolled 2007–2018 and followed until 2022	Age, gender, education, income, comorbidities, drug use, severity of diabetes
Sun et al., 2026 (21)	Several countries	TriNetX Global Collaborative Network	2005–2025	Retrospective cohort study	184,970	68484/107959,8527 cases without reported sex	58.67	ICD-10	20 years	Demographics, comorbidities, medications, laboratory indicators, smoking, drinking, environmental exposure
Tang et al., 2024 (23)	America	U.S. Medicare administrative data	2016–2020	Retrospective cohort study	89074	40334/48740	75.1	ICD-9/10	The median duration of follow-up was 1.54 years	Demographic characteristics, comorbidities, and co-medications
Sunnarborg et al., 2022 (22)	Finland	Nationwide register-based Finnish study	1999–2015	Case-control study	9951	5950/4001	73.55	Diagnosed by a neurologist based on the UKPDSBB criteria	The exposure period was at least three years	Age, gender, region, duration of diabetes, comorbidities
Rozani et al., 2024 (20)	Israel	The diabetes registration and drug purchase databases of Maccabi Healthcare Services	1999–2018	Retrospective cohort study	86229	46477/39752	60	Based on the algorithm for purchasing anti-Parkinson drugs	An average of 9.2 years	Age, sex, myocardial infarction, congestive heart failure, chronic obstructive pulmonary disease, cancer, and stroke

NR, not reported; ICD, International Classification of Diseases; GLP-1RAs, glucagon-like peptide-1 receptor agonists; BMI, Body Mass Index; HbA1c, Glycated Hemoglobin A1c.

**Table 2:** The quality of included studies.

Author (year)	Selection	Comparability	Outcome/Exposure	Total	Quality
Brauer et al., 2020 (18)	3	2	2	7	High
Gamborg et al., 2025 (19)	3	2	3	8	High
Sun et al., 2026 (21)	3	2	3	8	High
Tang et al., 2024 (23)	3	2	3	8	High
Sunnarborg et al., 2022 (22)	3	2	2	7	High
Rozani et al., 2024 (20)	3	2	3	8	High

### Meta-analysis

**Figure 2:**
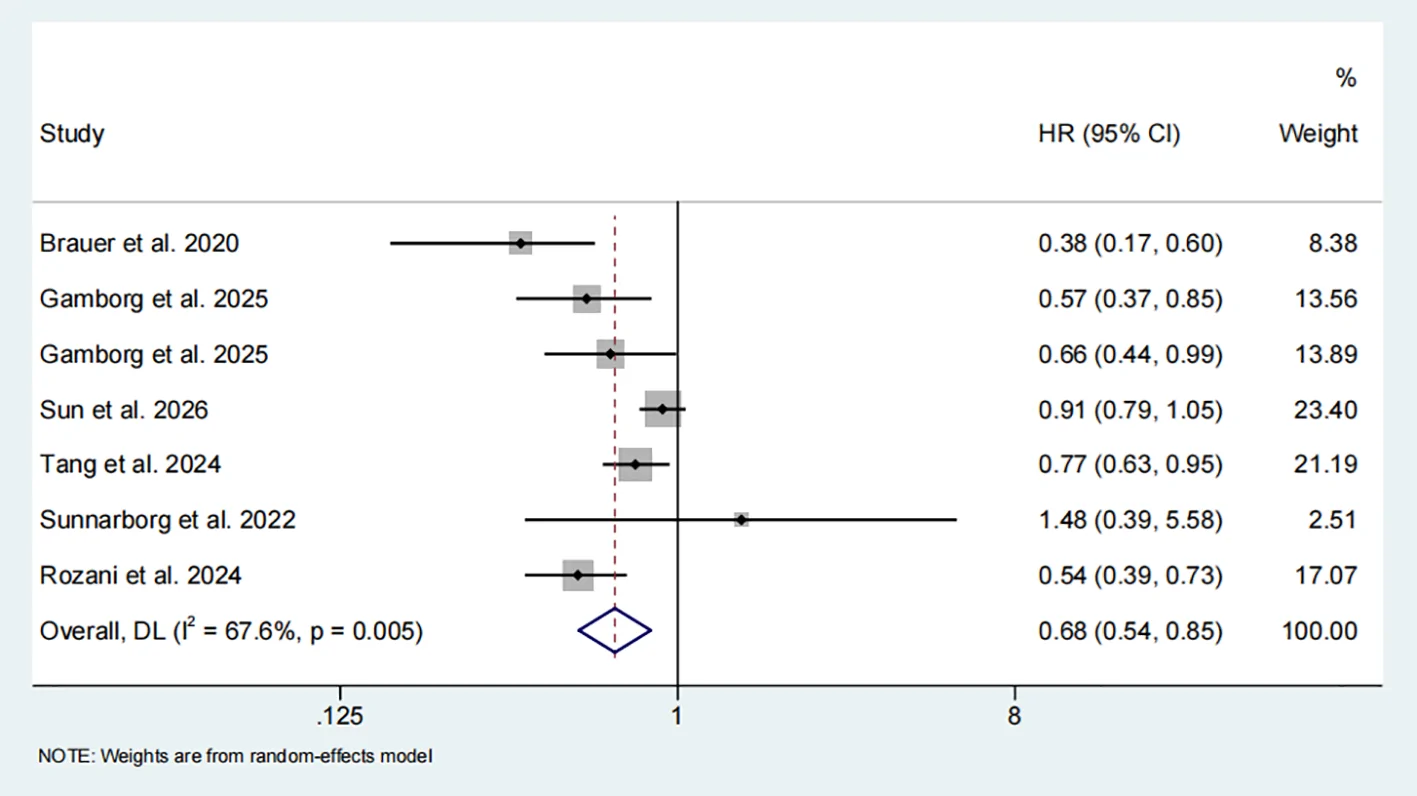
Forest plot of the meta-analysis for the association of Glucagon-like peptide-1 receptor agonists and the risk of Parkinson’s disease in patients with Type 2 Diabetes Mellitus. Each study is represented by a square (point estimate of hazard ratio, HR) and horizontal line (95% confidence interval, CI). The size of the square reflects the weight assigned to each study in the pooled analysis. The diamond represents the pooled HR using a random-effects model (due to significant heterogeneity, I² = 67.6%, P = 0.009). The overall pooled HR was 0.68 (95% CI 0.54–0.85, P = 0.001), indicating a statistically significant association between GLP-1RAs use and lower PD risk. HR < 1 favors GLP-1RAs. The analysis included 452,763 participants from six observational studies.

The present meta-analysis included six eligible studies involving a total of 452,763 participants. Significant between-study heterogeneity was detected (I² = 67.6%, P = 0.005), and thus the random-effects model was applied. The pooled results demonstrated that GLP-1RA exposure was significantly associated with a reduced risk of incident PD in patients with T2DM (HR = 0.68, 95%CI (0.54,0.85), P = 0.001) (**Figure 2**).

### Subgroup analysis

To explore potential sources of heterogeneity and further clarify the comparative neuroprotective effects of GLP-1RAs versus other hypoglycemic drugs, subgroup analyses were performed according to the types of comparator glucose-lowering drugs (**Table 3**).

**Table 3:** Subgroup analysis.

Subgroup analysis	n	Heterogeneity		HR(95%CI)	P Value
		I^2^	P		
Types of drugs					
GLP-1RAs vs DPP-4 inhibitor	2	38.10%	0.204	**0.60(0.43,0.83)**	**0.001**
GLP-1RAs vs Metformin	2	88.70%	0.003	**0.83(0.73,0.95)**	**0.006**
GLP-1RAs vs Other	2	49.9%	0.136	**0.73(0.60,0.87)**	**0.002**

GLP-1RAs, glucagon-like peptide-1 receptor agonists; DPP-4, dipeptidyl peptidase-4 inhibitor; HR, hazard ratio; CI, Confidence interval.

Bold text indicates that the differences are statistically significant.

Compared with DPP-4 inhibitors: Two studies were pooled for this subgroup. The analysis revealed that GLP-1RA exposure was associated with a significantly lower risk of PD in patients with T2DM compared to DPP-4 inhibitors (HR = 0.60, 95%CI: 0.43–0.83, P = 0.001).

Compared with metformin: Two studies compared GLP-1RAs with metformin. Patients with T2DM receiving GLP-1RAs had a significantly lower PD risk compared with those treated with metformin (HR = 0.83, 95% CI: 0.73–0.95, P = 0.006).

Compared with other hypoglycemic drugs (primarily sulfonylureas, insulin, thiazolidinediones, and meglitinides): Two studies compared GLP-1RAs with other hypoglycemic drugs. The pooled estimate indicated that GLP-1RA exposure was linked to a substantially lower risk of PD in patients with T2DM (HR = 0.73, 95% CI: 0.60–0.87, P = 0.002).

Given the limited number of studies included in each subgroup analysis, these findings should be interpreted as exploratory and hypothesis-generating rather than definitive comparative evidence.

### Sensitivity analysis

**Figure 3:**
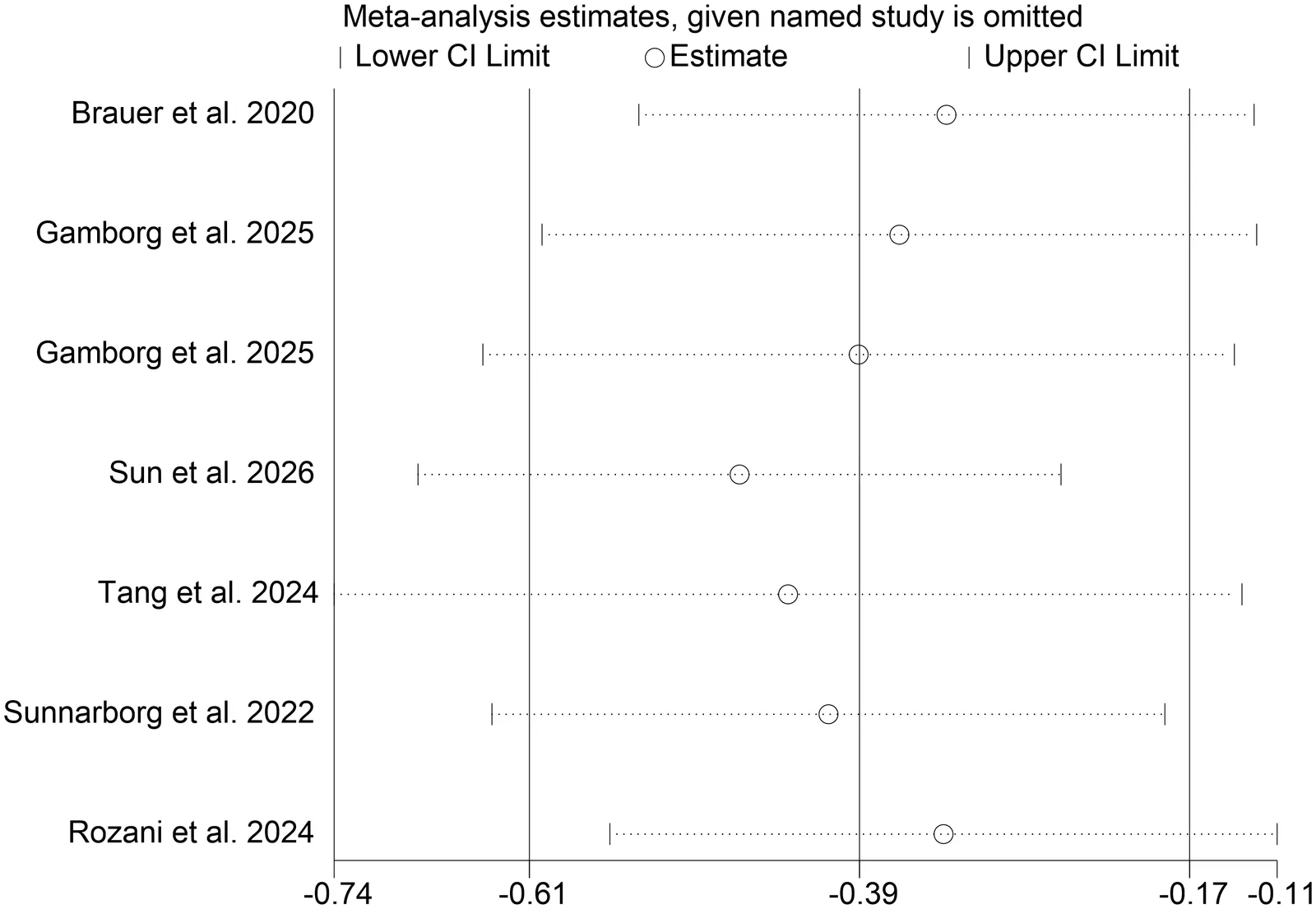
Sensitivity analysis of the association of Glucagon-like peptide-1 receptor agonists and the risk of Parkinson’s disease in patients with Type 2 Diabetes Mellitus. This analysis was performed to assess the robustness of the pooled estimate and to determine whether any single study disproportionately influenced the overall result. Each row represents the pooled HR (with 95% CI) calculated after excluding the corresponding study. The vertical dashed line indicates the overall pooled HR (0.68) from the full meta-analysis. The results remained stable across all iterations (range of HRs: approximately 0.65–0.72), with all 95% CIs not crossing 1.0, suggesting that no single study dominated the pooled estimate and that the overall finding was robust.

A leave-one-out sensitivity analysis was performed to evaluate the stability of the pooled results. The overall effect estimate remained quantitatively unchanged after the omission of any single study, demonstrating that our meta-analytic findings were statistically robust and not disproportionately influenced by any individual investigation (**Figure 3**).

### Publication bias

**Figure 4:**
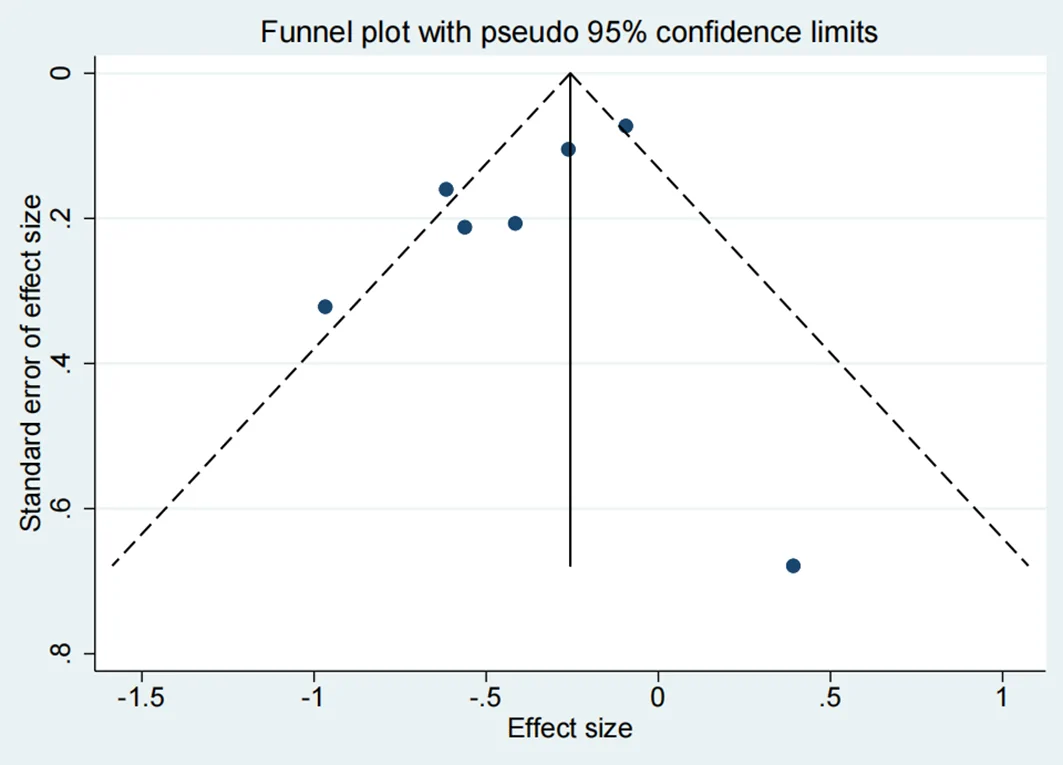
Funnel plot of the association of Glucagon-like peptide-1 receptor agonists and the risk of Parkinson’s disease in patients with Type 2 Diabetes Mellitus. Each point represents an individual study included in the meta-analysis, plotted with its effect estimate (log HR) on the horizontal axis against its standard error (SE) on the vertical axis. In the absence of publication bias, points should be symmetrically distributed around the pooled effect line (vertical dashed line).

Visual inspection of the funnel plot revealed slight asymmetry (**Figure 4**). However, Egger’s regression test indicated no statistically significant publication bias (P = 0.159).

## Discussion

This systematic review and meta-analysis of large-scale real-world datasets evaluated the association between GLP-1RA exposure and the risk of PD among patients with T2DM. Six eligible observational studies encompassing 452,763 participants were ultimately included. Our pooled analysis revealed that GLP-1RA exposure is significantly associated with a lower risk of PD in patients with T2DM. Subgroup analyses further confirmed that this protective inverse association remained robust when GLP-1RAs were compared with DPP-4 inhibitors, metformin, and other conventional hypoglycemic drugs. Collectively, these findings provide rigorous real-world evidence supporting the neuroprotective potential of GLP-1RAs and highlight their promising role as a disease-modifying strategy for PD, offering valuable implications for future drug repurposing investigations.

The present findings are consistent with exploratory results from several previous large-scale real-world cohort studies (18, 20, 23). Accumulating real-world evidence has indicated that GLP-1RA exposure is significantly associated with a lower risk of PD (18, 20, 23). Of note, recent meta-analyses of randomized controlled trials failed to demonstrate a statistically significant overall beneficial effect of GLP-1RAs on PD outcomes. Conversely, GLP-1RA treatment was found to increase the incidence of gastrointestinal adverse events in these trial-based analyses (27, 28).

This apparent discrepancy between those RCT-derived null findings and the reduced PD risk observed among individuals with T2DM in our meta-analysis warrants careful differentiated interpretation. First, the primary endpoint of our observational study was the risk of incident PD, corresponding to the preventive effect against PD onset, whereas RCTs mainly evaluated the disease-modifying efficacy of GLP-1RAs among patients with established PD, an outcome that reflects the therapeutic effect of these agents at distinct stages of the PD natural history. To date, no direct evidence confirms whether the risk-lowering effect of GLP-1RAs against incident PD in patients with T2DM can be extrapolated to slow disease progression in individuals already diagnosed with PD. Second, the relatively short follow-up duration (typically 12 months) and limited sample sizes of existing RCTs may undermine their statistical power to detect the slowly cumulative neuroprotective effects of GLP-1RAs. Accordingly, the observational protective association identified in our work is not scientifically contradictory to the neutral outcomes from previous RCTs; instead, these two sets of results indicate that dedicated study designs are required to separately explore the roles of GLP-1RAs in PD prevention and PD treatment. Future randomized controlled trials should be stratified according to disease stages (preventive intervention versus therapeutic treatment), and long-term prospective follow-up studies enrolling high-risk T2DM populations are essential to validate our research hypothesis.

The observational findings from the present meta-analysis can provide evidence-based references for the design and rationale of future large-scale, long-term RCTs, which remain indispensable for establishing causal inference. From a clinical perspective, our results offer valuable implications for hypoglycemic agent selection among patients with T2DM at high risk of developing PD. Although the identified inverse association supports the hypothesis that GLP-1RAs may exert neuroprotective effects in T2DM patients carrying additional PD risk factors, this assumption needs to be verified via prospective trials before any formal clinical recommendations can be formulated. Current available evidence is insufficient to support preferential prescription of GLP-1RAs for the sole purpose of PD prevention. Nevertheless, our observational results deliver preliminary suggestive evidence that merits further investigation into the preventive potential of GLP-1RAs against PD. These findings, however, cannot be regarded as definitive evidence to endorse the off-label use of GLP-1RAs for PD in routine clinical practice. Should off-label administration be considered in any setting, such practice must be implemented under stringent research protocols with rigorous ethical supervision.

Subgroup analyses in the present study demonstrated that GLP-1RA exposure was associated with a significantly lower risk of PD compared to DPP-4 inhibitors, metformin, and other hypoglycemic drugs. This observation carries important clinical implications, implying that the neuroprotective benefits conferred by GLP-1RAs are not merely attributable to optimized glycemic control but also stem from their unique intrinsic neuroprotective properties. DPP-4 inhibitors elevate endogenous GLP-1 concentrations indirectly by suppressing GLP-1 degradation, yet their pharmacological potency is substantially weaker than that of exogenous GLP-1RAs (29, 30), which may explain why its neuroprotective effects is relatively limited.

Metformin, the first-line glucose-lowering agent for type 2 diabetes mellitus, has been reported to exert potential neuroprotective effects in prior investigations (31). Nevertheless, our subgroup analysis identified that GLP-1RAs yielded stronger protective benefits against PD relative to metformin, further validating the independent neuroprotective role of GLP-1RAs. Comparisons versus other classes of glucose-lowering agents likewise reinforced the potential superiority of GLP-1RAs for PD prevention.

Given the limited number of primary studies included within each subgroup, estimates of between-study heterogeneity remain unstable, and these numerical intergroup differences should therefore not be overinterpreted. The observed disparities may reflect genuine pharmacological distinctions between GLP-1RAs and each comparator medication class; alternatively, they could originate from methodological variations across subgroups, including differences in study design, follow-up duration, baseline participant characteristics, and diagnostic approaches for incident PD. Accordingly, these subgroup findings should be regarded as exploratory analyses intended primarily to verify the consistency of the neuroprotective signal across distinct clinical settings, rather than to establish definitive hierarchical rankings of comparative efficacy.

The inverse association between GLP-1RA exposure and reduced risk of PD observed in our meta-analysis is biologically plausible given the well-established neuroprotective mechanisms of GLP-1RAs. GLP-1 receptors are broadly distributed throughout the central nervous system, especially in brain regions vulnerable to PD-related pathological lesions, including the substantia nigra, striatum, hippocampus, and cerebral cortex (32). Accumulating preclinical evidence has demonstrated that GLP-1R activation within these brain regions exerts anti-inflammatory, antioxidant and anti-apoptotic activities via multiple signaling cascades such as the cAMP/PKA, PI3K/Akt and MAPK pathways, ultimately preserving dopaminergic neurons from degeneration (15–17, 32).

The neuroprotective actions of GLP-1RAs against PD are partially mediated through modulation of the PI3K/Akt signaling pathway, whose key downstream targets include FoxO, mTOR and NF-κB (33). Upon ligand binding to GLP-1 receptors, this signaling cascade suppresses GSK-3β activity and mitigates the pathological deposition of neurotoxic proteins, most notably α-synuclein (33). GLP-1R activation also elevates intracellular cyclic adenosine monophosphate (cAMP) levels, which confers protective effects against PD pathology by triggering downstream signaling to dampen neuroinflammation and oxidative stress and inhibit neuronal apoptosis (34). In addition, GLP-1RAs promote autophagic flux to facilitate the clearance of α-synuclein and restrain its aberrant intracellular aggregation within neurons (35). Collectively, these molecular mechanisms underpin the biological rationale whereby GLP-1RAs may slow or halt progressive PD pathogenesis. Detailed mechanistic pathways are illustrated in the **Supplementary Material** (**Supplementary Figure 1**).

Several limitations of the present study should be acknowledged when interpreting the findings. First, all eligible studies included in our meta-analysis adopted an observational design. Although each primary study performed rigorous statistical adjustment for measurable confounders, including age, sex, baseline comorbidities, and concomitant medications, potential confounding effects from unmeasured or unknown factors could not be fully excluded. These unadjusted variables may encompass family history of PD, environmental toxin exposure, dietary habits, physical activity levels, and socioeconomic status. Importantly, GLP-1RA prescribing in routine clinical practice is subject to substantial indication bias, whereby treatment decisions are systematically influenced by BMI, cardiovascular risk profiles, and renal function. Since these clinical parameters may independently correlate with PD susceptibility, the observed reduction in PD risk among GLP-1RA users cannot be solely attributed to the pharmacological effects of GLP-1RAs. Therefore, our findings should be interpreted as exploratory associative evidence rather than definitive causal inference. Further validation based on rigorously designed randomized controlled trials or advanced causal analytical approaches, such as instrumental variable analysis and target trial emulation, are warranted in future studies.

Second, significant between-study heterogeneity was observed across the pooled analyses, with an extremely high heterogeneity level detected in the metformin comparison subgroup (I² = 88.7%). Such heterogeneity may stem from discrepancies in follow-up duration, PD diagnostic criteria, baseline population characteristics, and definitions of comparator agents across individual studies. Although the random-effects model was applied to synthesize pooled estimates and subgroup analyses were performed to explore potential sources of heterogeneity, the overall results should still be interpreted with caution.

Third, all participants enrolled in the original studies were exclusively patients with T2DM, which limits the generalizability of our conclusions. Accordingly, the observed protective association cannot be directly extrapolated to nondiabetic populations.

Fourth, several included studies had relatively modest sample sizes, and subgroup analyses stratified by individual GLP-1RA agents were not performed. This precluded a comprehensive evaluation of potential differential neuroprotective efficacy among distinct GLP-1RA subtypes.

Fifth, owing to the limited number of six included studies, the statistical power of Egger’s regression test for publication bias was constrained. The asymmetric distribution observed in the funnel plot should therefore be interpreted cautiously. Although both visual inspection and Egger’s test revealed no significant publication bias, we cannot completely rule out the possibility of small-study effects or underpublication of negative or null findings.

Finally, the protocol of this systematic review was not prospectively registered in public databases such as PROSPERO. Although the present study was strictly reported in accordance with the PRISMA 2020 guidelines, the absence of a registered protocol may introduce potential reporting bias, which should be considered when evaluating our results.

## Conclusion

In conclusion, this meta-analysis of large-scale real-world observational data demonstrates that GLP-1RA exposure is significantly associated with a reduced risk of PD among patients with T2DM. This protective association remained consistent across multiple classes of hypoglycemic drugs, supporting the hypothesis that GLP-1RAs confer promising neuroprotective potential worthy of further in-depth verification. Given the inherent limitations of observational studies and the inevitable residual confounding, the present findings should be interpreted as preliminary and hypothesis-generating rather than definitive causal evidence. Future large-scale, standardized prospective cohort studies and well-designed randomized controlled trials are urgently required to validate our results and definitively determine whether this observed protective association reflects a true causal neuroprotective effect of GLP-1RAs.

## Data Availability

The datasets presented in this study can be found in online repositories. The names of the repository/repositories and accession number(s) can be found in the article/**Supplementary Material**.
